# Preservation Method and Phosphate Buffered Saline Washing Affect the Acute Myeloid Leukemia Proteome

**DOI:** 10.3390/ijms19010296

**Published:** 2018-01-19

**Authors:** Rebecca Wangen, Elise Aasebø, Andrea Trentani, Stein-Ove Døskeland, Øystein Bruserud, Frode Selheim, Maria Hernandez-Valladares

**Affiliations:** 1 The Proteomics Unit at the University of Bergen, Department of Biomedicine, University of Bergen, Jonas Lies vei 91, 5020 Bergen, Norway; Rebecca.Wangen@uib.no (R.W.); Elise.Aasebo@uib.no (E.A.); Frode.Selheim@uib.no (F.S.); 2 Department of Molecular Biology, University of Bergen, Thormøhlensgate 55, 5008 Bergen, Norway; 3 Department of Clinical Science, University of Bergen, Jonas Lies vei 87, 5021 Bergen, Norway; Andrea.Trentani@uib.no (A.T.); Oystein.Bruserud@uib.no (Ø.B.); 4 Department of Biomedicine, University of Bergen, Jonas Lies vei 91, 5020 Bergen, Norway; Stein.Doskeland@uib.no

**Keywords:** proteomics, acute myeloid leukemia, preservation, phosphate buffered saline, dimethyl sulfoxide, mass spectrometry, sample preparation

## Abstract

Acute myeloid leukemia (AML) primary cells can be isolated from peripheral blood, suspended with media containing bovine serum and cryoprotectant, and stored in liquid nitrogen before being processed for proteomic analysis by mass spectrometry (MS). The presence of bovine serum and human blood proteins in AML samples can hamper the identifications of proteins, and thereby reduce the proteome coverage of the study. Herein, we have established the effect of phosphate buffered saline (PBS) washing on AML patient samples stored in media. Although PBS washes effectively removed serum and blood contaminants, the saline wash resulted in cell burst and remarkable protein material loss. We also compared different methods to preserve the AML proteome from THP-1 and Molm-13 cell lines before MS analysis: (1) stored in media containing bovine serum and dimethyl sulfoxide (DMSO); (2) stored as dried cell pellets; and (3) stored as cell lysates in 4% sodium dodecyl sulfate (SDS). MS analysis of differently preserved AML cell samples shows that preservation with DMSO produce a high number of fragile cells that will burst during freezing and thawing. Our studies encourage the use of alternative preservation methods for future MS analysis of the AML proteome.

## 1. Introduction

Acute myeloid leukemia (AML) is an aggressive hematopoietic cancer that is characterized by limited differentiation and uncontrolled proliferation of myeloid progenitor cells [1]. There is large heterogeneity among the patients regarding karyotype and genetic abnormalities, cellular phenotype, and prognosis after anti-leukemic treatment [1]. Evaluation of cytogenetic and mutational aberrations in these malignant cells is used for prognostication and treatment selection of AML patients [2], emphasizing the importance of cell biological characterization. In the years to come, it is expected that mass spectrometry (MS)-based protein quantification will play an essential role within diagnostics and personalized medicine in clinical laboratories [3], in addition to unraveling phenotypes and complex biological processes [4]. Applications of proteomics in AML, thus may add new understanding of the biological heterogeneity in AML. 

Within the field of MS-based proteomics, large efforts have been put into optimization of sample preparation workflows, including cell lysis and protein digestion [5,6], liquid chromatography (LC)-MS instrumentation and methodology [7,8,9], and bioinformatics tools [10,11]. Technical variations leading to inaccurate or non-reproducible protein quantification may occur during sample preparation and MS analysis, and stricter requirements to quality control during these experimental procedures are now on the agenda [12,13]. Moreover, contaminants that were introduced during sample collection and preparation and the effect of long-term storage on samples may induce alteration of the proteome and/or influence the MS-based protein quantification [5,14]. 

Primary AML cells may be obtained from the bone marrow aspirate or from the peripheral blood of patients with a high number (>80%) of circulating blasts [15,16]. After gradient separation for the removal of non-leukemic cells, the blast cells can either be processed immediately or cryopreserved in 10% dimethyl sulfoxide (DMSO), supplemented with 10–20% fetal bovine serum (FBS), and stored in liquid nitrogen for e.g., biobanking purposes. Cryopreserved samples can be used in a wide range of experiments, although this sample storage procedure might influence the outcome of MS-based phosphoproteomic studies [5]. The presence of abundant contaminants can challenge the proteomic identification and quantification of target proteins. In AML patient samples, remaining blood after the gradient separation and keratins from the lab workers represent a major source of non-AML protein contaminants. FBS can be considered as an additional protein contaminant when the samples are cryopreserved. The contaminating proteins should preferentially be removed prior to LC-MS analysis, as their corresponding peptides will compete with the peptides of interest during the MS measurement and suppress peptide signals of lower intensity [12].

In the current study, we have studied the effect of phosphate buffered saline (PBS) wash on cell pellets from AML patients and evaluate its impact on the AML proteome coverage and quantification. Furthermore, the effect of three storage and preparation protocols on two AML-derived cell lines: (1) liquid nitrogen storage in media containing bovine serum and DMSO; (2) liquid nitrogen storage as dried cell pellets; and (3) −80 °C storage as cell lysates in 4% sodium dodecyl sulfate (SDS) has been investigated for future applicability on the preservation of the AML primary cell material for MS analysis. 

## 2. Results

### 2.1. Effect of Phosphate Buffered Saline (PBS) Wash on Primary Acute Myeloid Leukemia (AML) Cells

**Figure 1:**
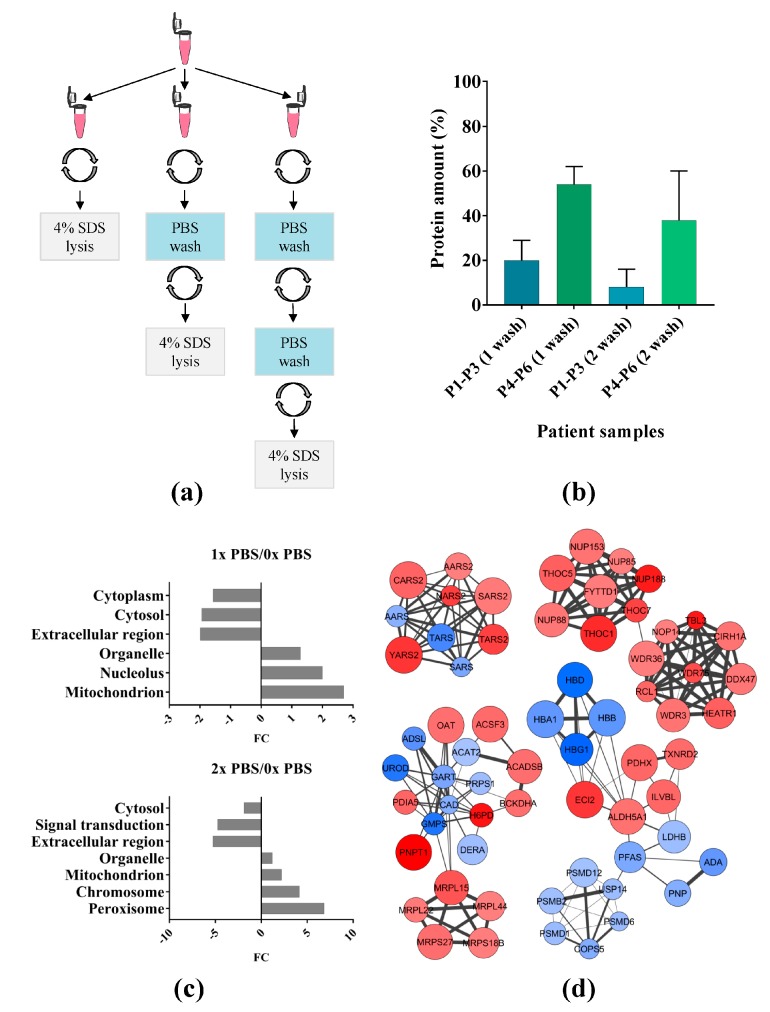
The effect of phosphate buffered saline (PBS) wash(es) on the acute myeloid leukemia (AML) proteome. (**a**) Experimental design including one and two PBS washes of the primary AML cell pellets; (**b**) Protein amounts observed in six patient samples after one and two PBS washes represented with the mean and standard deviation (SD). P1–P3 are patient samples containing approximately 8 million of primary cells each while P4–P6 patient samples contain higher amounts of primary cells (ranging from 8 to 30 million); (**c**) Enrichment analysis of gene ontology (GO) terms by a GO tool software using the GO slim search mode; (**d**) Major protein clusters identified with MCode. Protein nodes are colored with red when they are more abundant in samples washed with PBS once and with blue when they are more abundant in unwashed samples. Increased size of nodes reflects a more significant *p*-value and thicker edge width represents a more confident interaction from STRING analysis.

Cryopreserved AML patient samples in liquid nitrogen contain DMSO to protect the cells from rupture and serum in order to maintain cellular viability and function. Freezing medium containing 20% FBS and 10% DMSO is our standard for primary AML samples destined to cell culture assays, but should be removed prior to proteomic analysis [15,17]. Here, we used cryopreserved AML patient samples with different protein amount and different degree of blood contamination to explore the effect that PBS washing has on contaminant removal and on the AML cell proteome (Table S1 and Figure 1a). As visualized in Figure S1, one wash with PBS (1× PBS) efficiently reduced (~3 fold) the amount of bovine serum albumin and blood contamination from the patient samples. However, the protein amount was reduced by as much as 36–93% after only one washing step with PBS for the different patients (Figure 1b). For patient samples P1–P3 containing low amount of cells, the protein concentration was reduced by as much as 80 ± 9% (SD, standard deviation), while for patient samples P4–P6 containing a higher amount of cells, the reduction was considerable 46 ± 8% (SD). Moreover, a second PBS wash (2× PBS) even further reduced the protein amount in the samples. Thus, primary AML cells that have been stored in liquid nitrogen and 10% DMSO seem to become too fragile for PBS washes prior to sample processing for MS analysis. 

To examine the changes in the proteome after the PBS washing steps, we performed enrichment analyses with the web-based software a GO tool (Figure 1c). We found overrepresented proteins prior to 1× PBS wash denoted to cytoplasm, cytosol, and extracellular region, whereas the overrepresented proteins after 1× PBS were related to organelle, nucleolus, and mitochondrion. Similar enriched gene ontology (GO) terms were observed for primary AML cells after two PBS washes. The identified proteins for all of the significantly enriched GO terms are listed in Table S2. Protein interacting clusters affected after one PBS wash are shown in Figure 1d. Several t-RNA ligases, nuclear pore complex proteins, suppressors of the transcriptional defects of hpr1 delta by overexpression (THO) complex subunits, tryptophan-aspartic acid (WD) repeat-containing proteins, and mitochondrial ribosomal proteins were significantly more abundant after one PBS wash, while hemoglobin subunits and proteasome non-ATPase regulatory subunits were significantly more abundant when no PBS wash was carried out.

### 2.2. Effect of Sample Preservation for the Study of the AML Proteome

**Figure 2:**
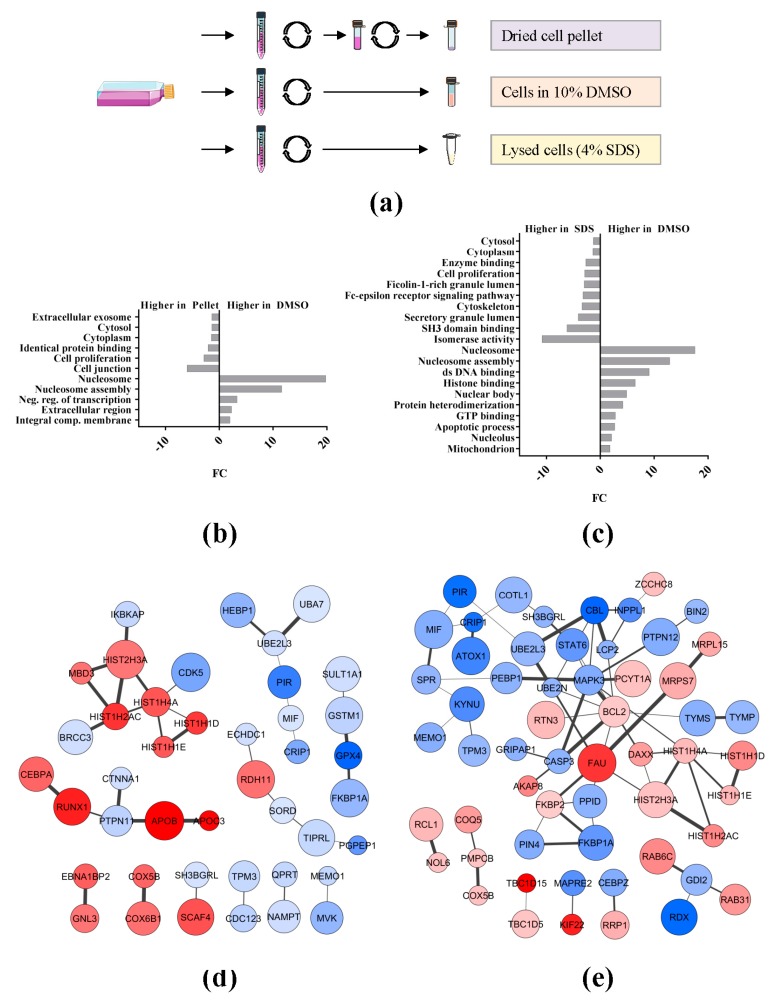
Proteomic outputs from THP-1 samples differently preserved. (**a**) Experimental design including the three preservation methodologies of the cell line proteome: as a dried pellet (pellet), in freezing media containing fetal bovine serum (FBS) and dimethyl sulfoxide (DMSO) and as a cell lysate in 4% sodium dodecyl sulfate (SDS); (**b**,**c**) Enrichment analysis of GO terms by the a GO tool software (GO slim search mode) using the significantly regulated proteins observed in the DMSO vs. pellet and in the DMSO vs. SDS comparisons, respectively; (**d**,**e**) Protein clusters identified with STRING using regulated proteins from the DMSO vs. pellet and from the DMSO vs. SDS comparisons, respectively. Protein nodes are colored with red when they are more abundant in the DMSO condition and with blue when they are more abundant in the pellet or SDS conditions. Increased size of nodes reflects a more significant *p*-value and thicker edge width represents a more confident interaction from STRING analysis.

We have previously demonstrated a significant difference in expression levels of the AML patient proteome between cryopreserved (DMSO samples from now onwards) and freshly processed cells (SDS samples from now onwards), i.e., lysed in 4% SDS directly after blast isolation [5]. Here, we expand our comparisons of different methods to preserve the AML proteins from THP-1 and Molm-13 cell lines by including the direct cell pellet storage in liquid nitrogen (pellet samples from now onwards) (Figure 2a).

We found similar number of quantified proteins in THP-1 and Molm-13 samples subjected to different storage conditions (Figure S2). More than 95% of the THP-1 and Molm-13 proteins was identified in all of the three preservation methods (Figure S3). The significantly regulated proteins with higher and lower protein abundance (as obtained from *t*- and *Z*-tests (*p*-value ≤ 0.05)), when comparing DMSO vs. pellet, DMSO vs. SDS, and pellet vs. SDS samples, were analyzed with the WebGestalt enrichment web tool and the GO Slim classification (Tables S3 and S4, Figure S4). By comparing the biological process categories of proteins with higher abundance in SDS (negative fold change, FC, DMSO/SDS) and of proteins with higher abundance in DMSO (positive FC DMSO/SDS) in the THP-1 cell line, we found proteins enriched in the SDS samples to be associated with response to stimulus (~2 fold), cell communication (~2.5 fold) and proliferation (~3 fold). The cellular component categories that were most enriched in SDS when compared to DMSO samples were associated to cytosol (~4 fold), whereas dominating protein classes with higher abundance in DMSO than SDS samples were found to be associated with nucleus (~1.5 fold) and mitochondrion (~2.5 fold). The enrichment analysis of DMSO vs. pellet samples showed approximately the same results for biological processes and cellular components as for the DMSO vs. SDS samples [18]. We found minor or no differences for the enrichment analyses of the pellet vs. SDS samples.

In order to further explore the significantly enriched GO terms and proteins in the DMSO compared to pellet and SDS samples, we performed new enrichment analyses with the web-based software a GO tool. As shown in Figure 2b,c, numerous significantly overrepresented GO terms were observed for proteins with higher abundance in DMSO samples (positive FC DMSO/pellet; positive FC DMSO/SDS). These include nucleosome, nucleosome assembly, negative transcription regulators, ds DNA binding, and membrane-bound organelles, like nucleus and mitochondrion. The significantly enriched proteins for both pellet and SDS samples were associated to cytosol and cytoplasm, and to cell proliferation. Other cytosol-related GO biological pathway terms with higher abundance in pellet or SDS samples included cell junction, cytoskeleton, SH3 domain binding, and FC-epsilon receptor signaling pathway. Thus, several of the GO terms observed for the pellet and SDS storage conditions have close relationships and harbor several common proteins. The identified proteins for each significantly enriched GO term in the differently preserved samples are listed in Table S5.

Clusters of interacting proteins with a higher abundance in the DMSO than in the pellet THP-1 samples consisted of several histones and AML biomarkers, such as CCAAT/enhancer-binding protein α (CEBPA) and Runt-related transcription factor 1 (RUNX1) transcription factors (Figure 2d). While histones appeared more abundant in DMSO than in SDS THP-1 samples, an E3 ubiquitin-protein ligase CBL (CBL) interacting protein cluster connected to cell surface receptor-triggered signaling pathways was less abundant in the DMSO samples (Figure 2e). 

When we compared the FC of proteins significantly regulated in the DMSO vs. SDS samples of the THP-1 cell line to AML patient samples, previously quantified by our group according to the stable isotope labeling with amino acids in cell culture (SILAC) and label-free procedures [5], we obtained good Spearman correlation values of 0.63 and 0.76, respectively (Figure S5). Thus, the effects of these different preservation methods on the THP-1 and patient proteomes are comparatively similar.

A similar WebGestalt GO enrichment to the one obtained with THP-1 samples was observed when comparing the most abundant proteins in the SDS and DMSO Molm-13 samples (Figure S6).

## 3. Discussion

### 3.1. PBS Wash

Here, we demonstrate that frozen/thawed primary AML cells should not be washed with PBS after liquid nitrogen storage. An extensive reduction of the protein amount was observed after washing the cell pellet with PBS. Several contaminants (i.e., FBS from the media), but also fragile cells (both leukemic and red blood cells) were lost during the first wash. Furthermore, the cells surviving the second wash are probably the most robust cells and/or subpopulation of leukemic cells from the first wash. We found a higher loss of proteins in samples with low cell number, which indicates that 1× PBS usage is not suitable for AML samples with low protein amounts. However, samples containing a higher number of cells appear to better resist the PBS-induced bursting. Moreover, the protein loss during the PBS wash might restrict phosphoproteomic sample preparations, which require higher amount of proteins. Enrichment analysis showed overrepresented proteins associated to organelle, nucleus, and mitochondrion after one wash, and chromosome, organelle, peroxisome, and mitochondrion after two washes. This suggested a cell bursting, presumably due to the osmotic shock produced when cells are placed abruptly in buffer and to cell susceptibility after exposure to PBS, altogether affecting subsequent AML proteome analysis.

### 3.2. Preservation Conditions

We have previously evaluated the freezing effect on the proteome of AML patients. We observed that mitochondrial proteins involved in the respiratory chain transport and proteins involved in mRNA splicing were more abundant in samples frozen with media containing 10% DMSO than in samples lysed with 4% SDS before freezing [5]. In order to further investigate this freezing effect and the consequences of preserving the AML proteome as a dried cell pellet additionally, we studied proteomic profiles in cell line samples preserved using these three different methodologies.

The comparison of the different preservation conditions of THP-1 and Molm-13 revealed that the number of quantified proteins in the samples frozen in 4% SDS at −80 °C, frozen as dried pellets, and frozen with media containing 10% DMSO was very similar. A functional enrichment analysis of the significantly expressed proteins found in DMSO vs. SDS or DMSO vs. pellet shows similar enrichment in the categories of biological processes and cellular components. This was expected as both SDS and pellet samples were, respectively, lysed and frozen immediately. The enrichment analysis of proteins more abundant in the DMSO samples showed proteins associated to mitochondrion and nucleus, which probably reflects cell bursting during freezing, thawing, and centrifugation of leukemic cells, as we have previously reported. Moreover, the expression of AML protein biomarkers, such as CEBPA and RUNX1, appeared to be influenced by preservation methodologies [19]. 

AML cells preserved in DMSO/FBS-containing media are used for cell culturing and flow cytometry assays. The highest leukocyte viability has been observed in patient samples cryopreserved in the presence of 4–5% DMSO [20]. This might encourage the use of reduced amounts of DMSO in the freezing media of AML cells of new biobanks. Here, we have shown that preservation methods as both dried pellet and as cell lysate in SDS alter the AML proteome minimally and are more suitable for proteomic studies. Although we did not study phosphoproteomic profiles in the dried pellet samples, our proteomics results incite the use of this preservation method to investigate the AML phosphoproteome.

## 4. Materials and Methods

### 4.1. Primary AML Cells

AML patient samples were collected, after written informed consent, from subjects with high peripheral blood blast counts (>10 × 10^9^/L). At least 95% of circulating mononuclear cells being leukemic blasts after a density gradient separation (Lymphoprep, Axis-Shield Density Gradient Media, Oslo, Norway) were stored in liquid nitrogen [21]. The AML biobank was registered at the Norwegian data protection office (reference 02/1118-5, 22 October 2002) and the presented studies here were approved by the Regional Ethics Committee III, University of Bergen, Norway (REK Vest 2013/634, 19 March 2013; 2015/1410, 19 June 2015; 2017/305, 14 February 2017). 

Six patient samples from the biobank, containing different amounts of red blood cell (RBC) contaminations after the density gradient separation, were selected for the study of PBS wash (1× solution) of AML primary cells (Table S1). After thawing on ice, the samples were split into two or three identical aliquots and centrifuged at 170× *g* at 4 °C for 5 min. The aliquots were unwashed or subjected to one or two PBS washes at 4 °C before lysis with 4% SDS in 0.1 M Tris·HCl pH 7.6 buffer, as shown in Figure 1a. Samples were kept at −80 °C until use.

### 4.2. AML Cell Lines

Two AML cell lines, THP-1 and Molm-13, were selected for the study of different sample preservation methodologies. The cells were cultured with 10% fetal calf serum (FCS) in a medium based on RPMI, with some metabolites adjusted to better reflect the concentrations encountered in circulating human plasma. Thus, alanine was 0.25 mM (0 in RPMI), glutamine was 0.5 mM (2.5 mM in RPMI), and glucose was 5 mM (11 mM in RPMI). The proline concentration was adjusted to 0.12 mM to reflect the level reported in leukemic bone marrow [22]. A low concentration (2 mM) of HEPES was present to help preservation of the pH at 7.4. The incubation was at 3% O_2_ and 6.5% CO_2_. Cells were split into three identical aliquots and spun at 140× *g* at RT for 5 min. One aliquot was lysed with 4% SDS in 0.1 M Tris·HCl pH 7.6 buffer and denatured at 95 °C for 7 min before storage at −80 °C, while another one was resuspended in RPMI media containing 20% FBS and 10% DMSO and frozen at −80 °C for 24–48 h before preservation in liquid nitrogen as previously described [5]. The remaining aliquot, as a dried cell pellet, was frozen and preserved in liquid nitrogen. Cell samples stored in DMSO-containing media and as dried pellets in liquid nitrogen were thawed on ice. After media removal from the DMSO-preserved samples by spinning at 170× *g* at 4 °C for 5 min, both sets of samples were lysed with 4% SDS in 0.1 M Tris·HCl pH 7.6 buffer.

### 4.3. Sample Preparation for Mass Spectrometry (MS) Analysis

SDS-lysed patient and cell line samples were processed and digested according to the filter-aided sample preparation (FASP) method [23,24]. All of the filter-processed samples used 20 μg of protein material. Peptides from both patient and cell line samples were cleaned up with the Oasis HLB μElution (Waters, Milford, MA, USA) protocol.4.4. Liquid Chromatography (LC)-MS Analysis.

Dried peptides were dissolved in 20 μL of 2% acetonitrile (ACN) and 0.5% formic acid (FA). Differently preserved THP-1 and Molm-13 samples were analyzed on an Orbitrap Elite mass spectrometer equipped with a nanospray Flex ion source coupled to an Ultimate 3000 Rapid Separation LC system (both from Thermo Scientific, Waltham, MA, USA). Approximately 0.5 μg peptides were pre-concentrated and separated, as previously described [5]. Patient samples without or with PBS wash(es) were analyzed on a Q Exactive HF Orbitrap mass spectrometer equipped with an Easy-Spray (Thermo Scientific) coupled to an Ultimate 3000 Rapid Separation LC system. Approximately 0.6 μg peptides were pre-concentrated on a 2 cm × 75 µm ID Acclaim PepMap 100 trapping column and separated on a 50 cm × 75 µm ID EASY-spray PepMap RSLC analytical column (both from Thermo Scientific). Bound peptides were eluted within a 195 min run using a binary gradient with buffer A (0.1% FA in water) and buffer B (0.1% FA in ACN).

### 4.4. Bioinformatic Analysis of Proteomic Data

Raw data were processed with MaxQuant version 1.5.5.1 [25,26]. The mass spectra were searched against concatenated reverse-decoy Swiss-Prot Homo sapiens database version 2017 02 (20172 entries) using the Andromeda search engine [27]. Label-free quantification was set to the LFQ mode and the LFQ minimal ratio count was one. Cysteine carbamidomethylation was used as a fixed modification; methionine oxidation and protein N-terminal acetylation as variable modifications. Trypsin was used as digestion protease and two missed cleavages were allowed. The false discovery rate was set at 0.01 for peptides and proteins; and, the minimum peptide length allowed was six amino acids. The proteomics quality control software PTXQC was used to check LC-MS data quality from output files generated by MaxQuant [28]. The Perseus 1.5.6.0 platform was used to analyze and visualize the protein groups obtained by MaxQuant [29]. 

Paired *t*-tests and *Z*-statistics, both run in Microsoft Excel, were applied to compare groups for statistical differences and to obtain fold change significance, respectively [30]. Functional enrichment analysis was performed by using the a GO tool and the web-based gene set analysis tool kit (WebGestalt) [31,32]. The manual calculation of the enrichment fold of the WebGestalt results is showed with an example (“all proteins” refers to the proteins uploaded in WebGestalt):

FC_pellet/DMSO_ = ((protein category_pellet_/all proteins_pellet_)/(protein category_DMSO_/all proteins_DMSO_))

Regulated proteins were imported into StringDB software version 10.5 for the analysis of protein interactions [33]. Protein networks were imported to Cytoscape version 3.3.0 [34]. The MCode plugin was used to identify highly interconnected networks in the PBS experiments [35].

Scatter plots and Spearman correlation were done using with GraphPad Prism v7.03 (GraphPad Software).

The raw MS data files of both the PBS wash and preservation experiments are available via ProteomeXchange with identifier PXD008361.

## 5. Conclusions

The objective of this study was to select optimized preparation conditions of AML samples for MS-based proteomic studies. The use of PBS wash for media and blood contaminant removal showed a highly modified proteome, especially for samples with low cell amounts. Enrichment analysis showed overrepresented proteins that were associated to organelle, nucleus, and mitochondrion, suggesting cell burst after the PBS washing step. The current use of 20% FBS/10% DMSO in the freezing medium of our AML cell line samples affect the quantification of AML proteins when compared to samples lysed and stored in 4% SDS and to samples stored as a dried pellet. We found several proteins with GO terms involved in cell proliferation, regulation of phosphorylation and signal transduction underrepresented in samples cryopreserved in 20% FBS/10% DMSO when compared to dried pellet and 4% SDS storage conditions. A universal preservation method of the AML samples cannot be selected for all subsequent cell-based, genomics and proteomics studies. However, a fraction of the AML blasts after isolation could be immediately frozen as a dried cell pellet or lysed with 4% SDS for future MS-based proteomic characterization. 

## Supplementary Materials

Supplementary materials can be found at www.mdpi.com/1422-0067/19/1/296/s1.

## Abbreviations

ACN	Acetonitrile
AML	Acute myeloid leukemia
DMSO	Dimethyl sulfoxide
FA	Formic acid
FASP	Filter-aided sample preparation
FBS	Fetal bovine serum
FCS	Fetal calf serum
FC	Fold change
GO	Gene ontology
LC	Liquid chromatography
MS	Mass spectrometry
PBS	Phosphate buffered saline
RBC	Red blood cell
SD	Standard deviation
SDS	Sodium dodecyl sulfate
SILAC	Stable isotope labeling with amino acids in cell culture
