# Supplementary material for: Preservation Method and Phosphate Buffered Saline Washing Affect the Acute Myeloid Leukemia Proteome
Source: Int J Mol Sci. 2018 Jan 19;19(1):296. doi: 10.3390/ijms19010296 (PMC5796241; doi:10.3390/ijms19010296)
Supplement: Supplementary file 1 [file ijms-19-00296-s001.zip › Figures and Tables.pdf]

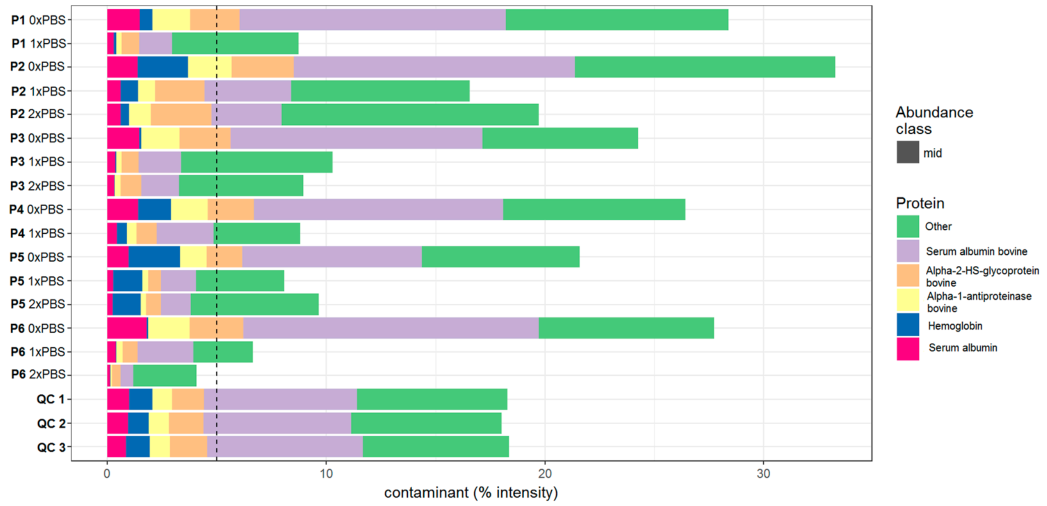

Figure S1. Intensity percentage of contaminants observed in primary AML samples before and after PBS wash(es).The figure is obtained with the PTXQC software (see Materials and Methods on the main text). Protein contaminants are listed on the right. The purple, orange and yellow bars indicate bovine proteins from the FBS in the media, while the blue and pink bars indicate human proteins highly abundant in blood. QC1-3 are pools of P1–P6 samples.

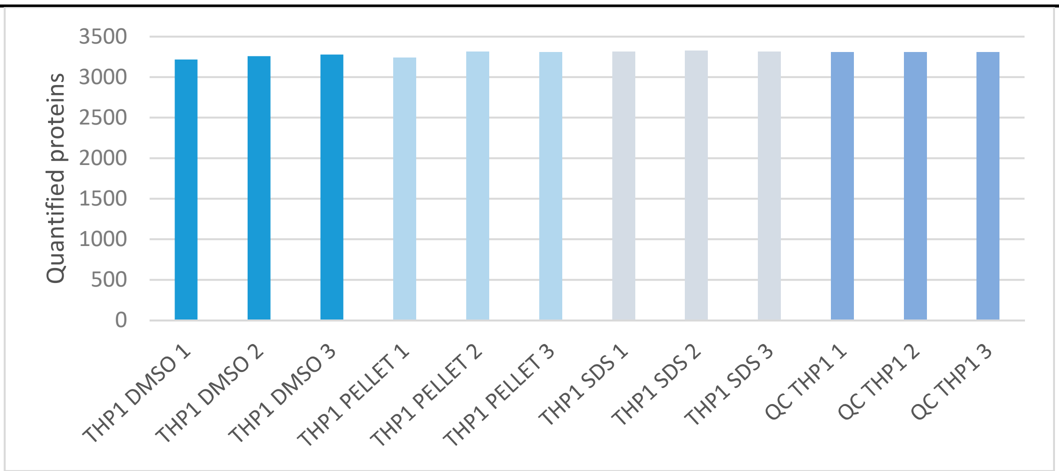

(**a**)

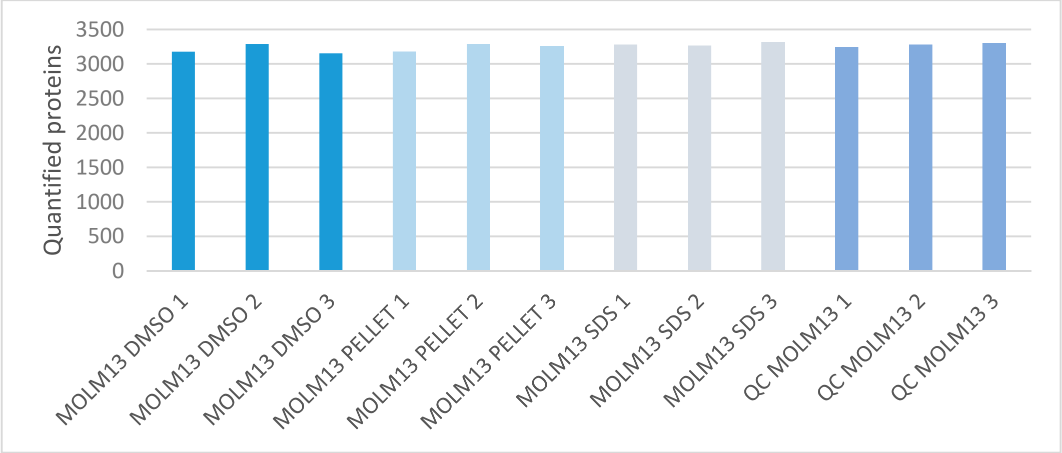

(**b**)

Figure S2. Number of quantified proteins from the different preservation conditions. The number of quantified proteins by mass spectrometry (MS) analyses of the differently preserved samples prepared by the filter-aided sample preparation (FASP) methodology. (**a**) Shows triplicates of each preservation method for the THP-1 cell line samples and (**b**) for the Molm-13 samples. QC samples are a pool of the replicates for each of the cell line samples.

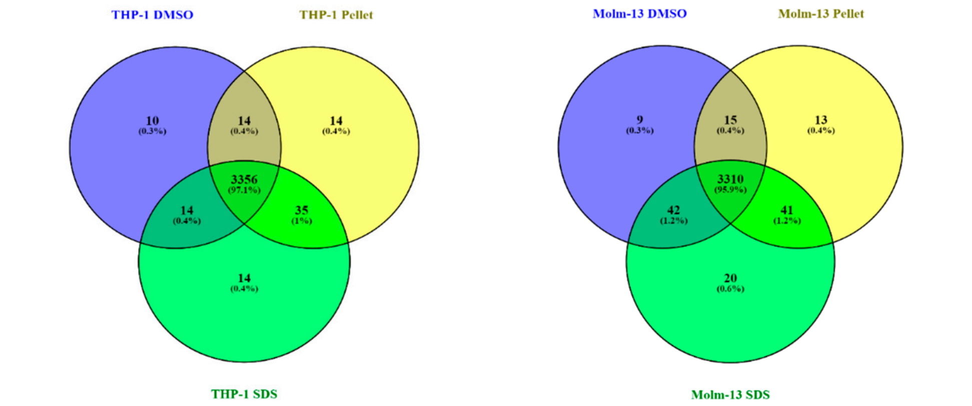

(**a**) (**b**)

Figure S3. Venn diagrams of the MS-quantified proteins obtained from the three preservation methods. Venn diagrams from proteins quantified in two out of the three replicates of each preservation condition for (**a**) THP-1 and (**b**) Molm-13 were performed using Venny 2.1 software (http://bioinfogp.cnb.csic.es/tools/venny/index.html).

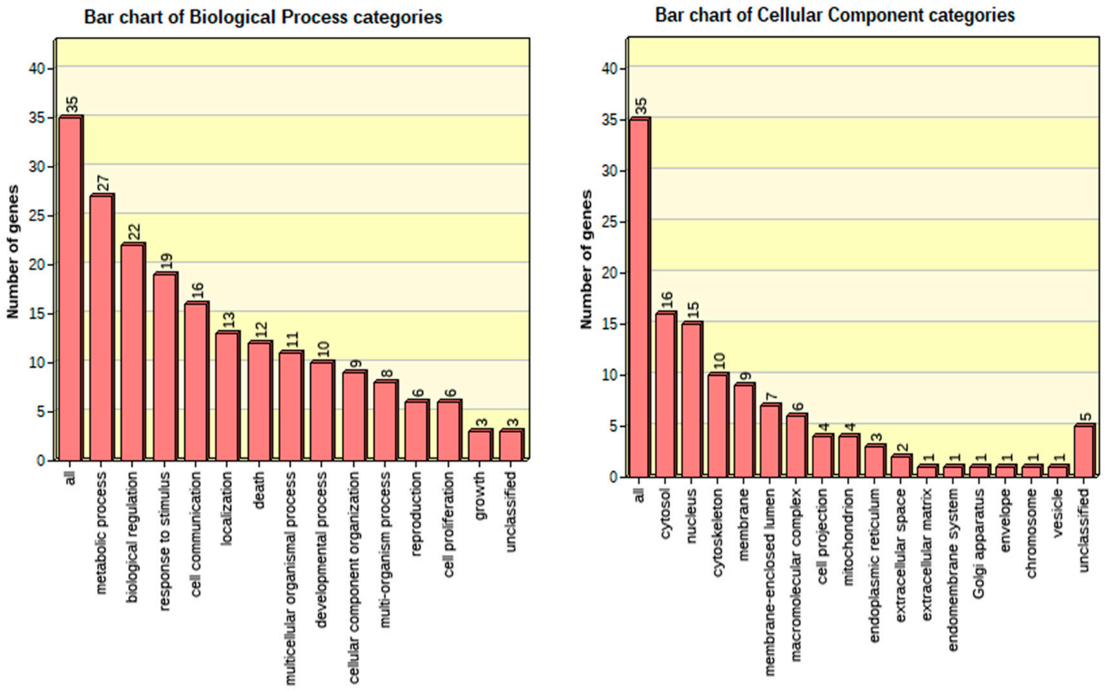

(**a**) with a negative fold change DMSO/SDS

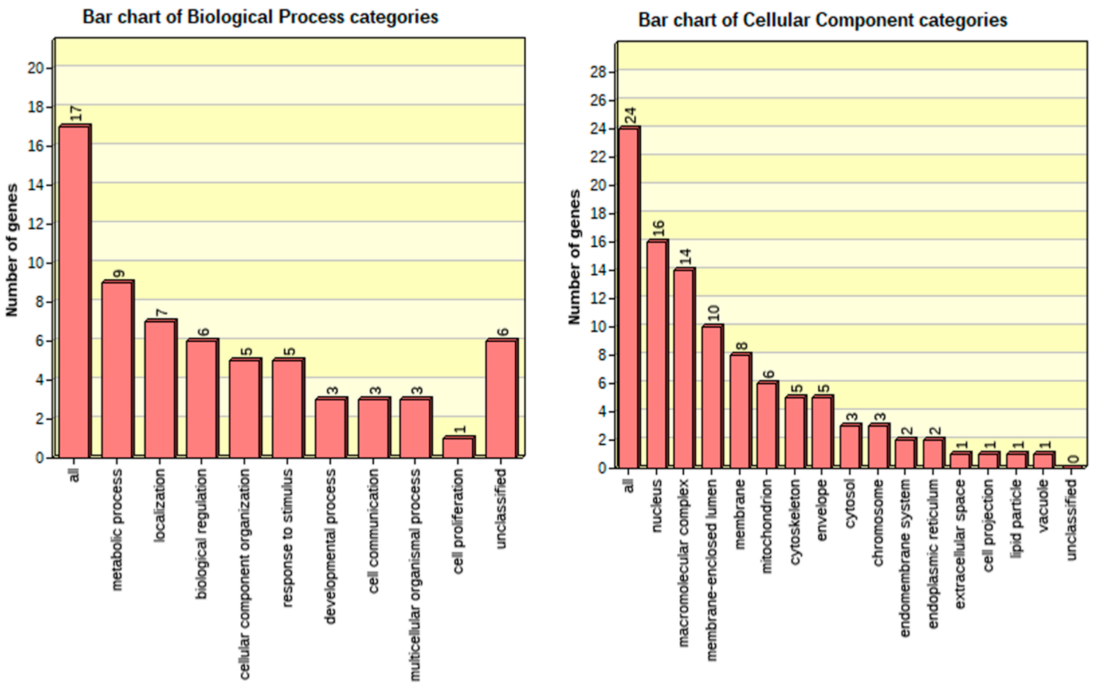

(**b**) with a positive fold change DMSO/SDS

Figure S4. GO enrichment of regulated proteins in THP-1 samples. The enrichments were performed with the WebGestalt tool using the GO slim classification.

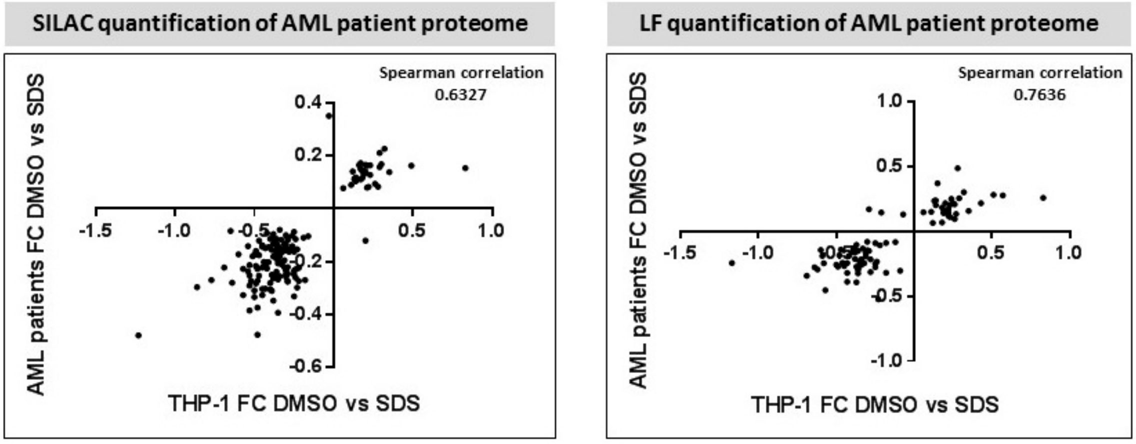

Figure S5. Correlation between the fold changes (FC) of 159 and 87 proteins found significantly regulated in the THP-1 and patient sample DMSO vs. SDS comparison when using stable isotope labeling with amino acids in cell culture (SILAC) (left plot) and label-free (LF) quantification (right plot) of the patient proteome, respectively. Plots and calculation of the Spearman correlation values were done using GraphPad Prism.

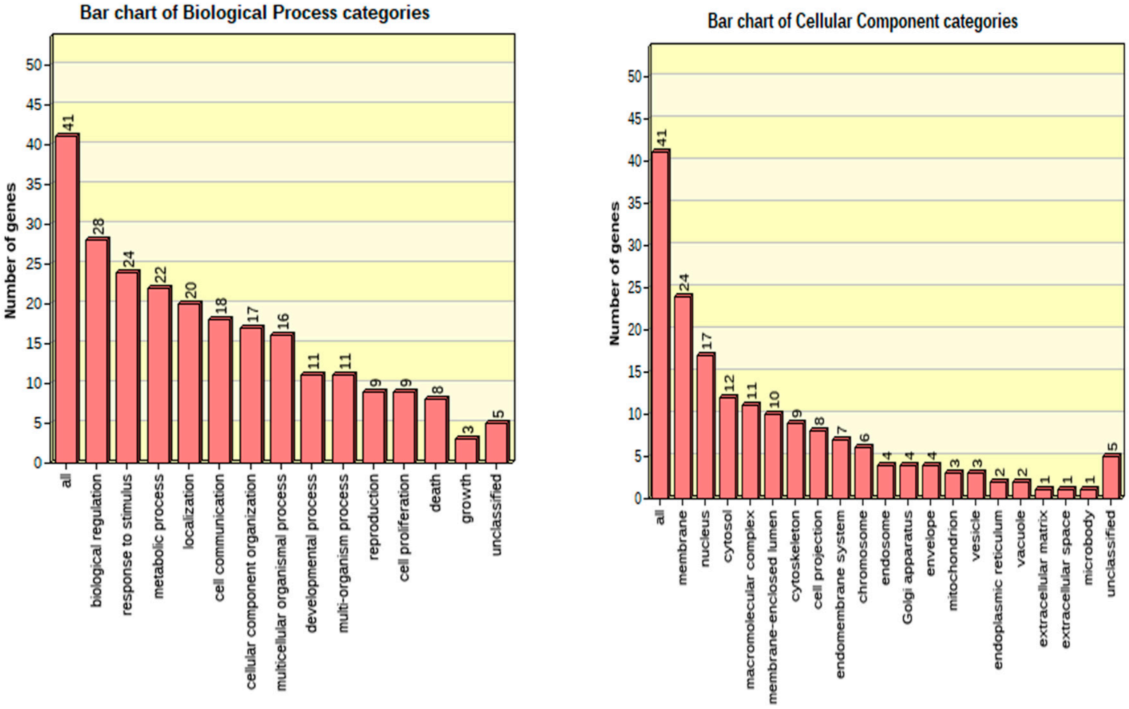

(**a**) with a negative fold change DMSO/SDS

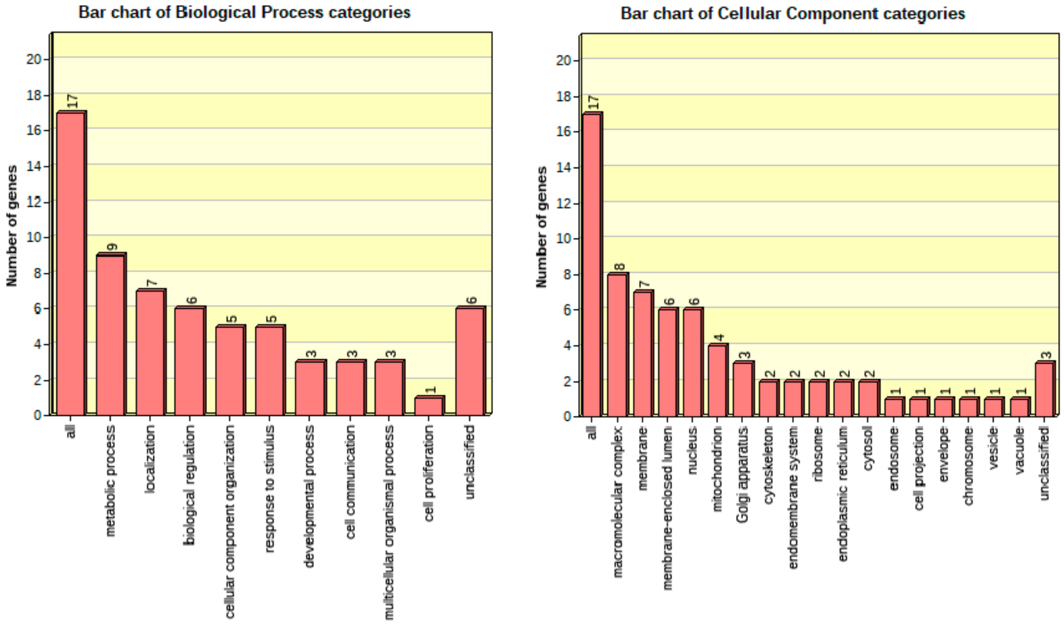

(**b**) with a positive fold change DMSO/SDS

Figure S6. GO enrichment of regulated proteins in Molm-13 samples. The enrichments were performed with the WebGestalt tool using the GO slim classification.

Table S1. AML patient samples used in the PBS wash experiment.

Patient/Million of Cells Per Sample	Pellet Description [a]	0 Wash (ug/uL) [b]	1st PBS Wash (ug/uL) [b]	2nd PBS Wash (ug/uL) [b]
P1/8	Moderate RBC contamination	0.48	0.12	-
P2/ND	High RBC contamination	0.53	0.04	-0.01
P3/8	Minimal RBC contamination	0.82	0.23	0.12
P4/8	High RBC contamination	0.94	0.49	-
P5/30	High RBC contamination	1.49	0.64	0.17
P6/10	No RBC contamination	0.96	0.61	0.52

[a] Red blood cell (RBC) contamination of AML cell pellets are assessed after centrifugation. Moderate RBC contamination represents traces of RBC observed in the white AML primary cell pellets. High RBC contamination represents red cell pellets, which contain AML primary cells and a high number of RBC. [b] Protein concentrations before and after PBS washes are determined by the BCA assay (Thermo Scientific, Waltham, MA, USA). ND stands for not determined.

Table S3. Number of significantly regulated protein groups observed when comparing two preservation methods.

Method Comparison	Molm-13	THP-1
DMSO vs. Pellet	↑62, ↓49 (111)	↑40, ↓47 (87)
DMSO vs. SDS	↑17, ↓43 (60)	↑47, ↓59 (106)
Pellet vs. SDS	↑16, ↓75 (91)	↑23, ↓43 (66)

↑ Positive fold change; ↓ Negative fold change. Total significant protein groups between parentheses.

Table S2. GO enrichment from a GO tool of proteins up- and down-regulated after one and two PBS washes. Uploaded separately.

Table S4. List of THP-1 and Molm-13 proteins significantly regulated in the three preservation methods. Uploaded separately.

Table S5. GO enrichment from a GO tool of THP-1 proteins more abundant in the three preservation conditions. Uploaded separately.
